# Emerging trends in the fabrication and applications of plant virus-gold nanoparticle hybrids: A comprehensive review

**DOI:** 10.1016/j.mtbio.2025.102237

**Published:** 2025-08-27

**Authors:** Haziq Naseer Khan, Nguyêt-Thanh Ha-Duong

**Affiliations:** Université Paris Cité, CNRS, ITODYS, F-75013, Paris, France

**Keywords:** Plasmonic, Assembly, Mineralization, Grafting, Genetic mutation

## Abstract

Plant viruses exhibit a diverse range of morphologies, including rod-shaped and icosahedral forms. Many of these viruses are detected and treated by the plasmonic nanoparticles of both silver and gold. In recent years, there has been an increasing interest in the use of these viruses as bio-templates for the synthesis of biohybrid materials using plasmonic nanomaterials, particularly gold nanoparticles (AuNPs). This approach aims to explore the unique properties of these Nano-hybrids to focus their applications on biomedicine, catalysis, and biosensing. This review focuses on various synthesis methods of these nanohybrids. Special attention is given to how virus morphology, nanoparticle shape, and surface chemistry affect the synthesis and stability of these nanohybrids. Additionally, the influence of genetic modifications to coat proteins is highlighted as a means to enhance nanoparticle binding and selectivity. Furthermore, the review discusses the prospective benefits and future directions for research on virus-based nanohybrids.

## Hightlights

• Grafting Methods: Different techniques attach AuNPs to virus-based scaffolds.
• Genetic modifications to improve control over nucleation and structural stability.
• Shape & Composition effects of AuNP tailor optical and catalytic properties.
• Applications of NanoHybrids for SERS, CT, and PTT therapies.
• Future Applications: Virus scaffolds in sensing, batteries, therapeutic devices.

## Graphical abstract

**Figure:**
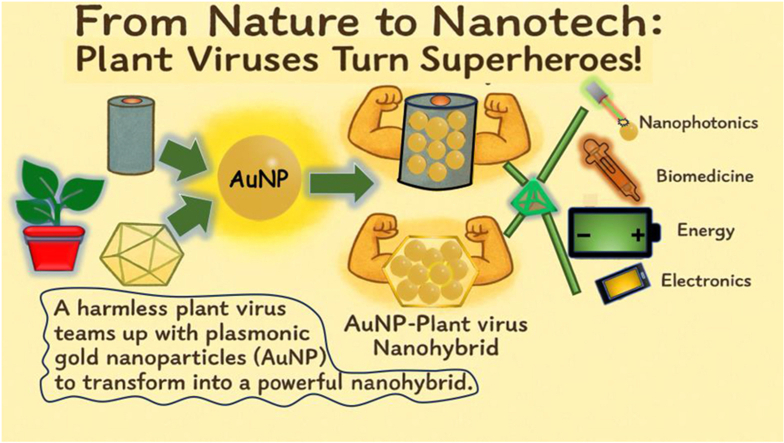

**Table:** Abbreviations

Analytical enhancement factor	AEF
Gold Nanoparticle	AuNP
Biotemplated lithography of inorganic nanostructures	BLIN
Brome mosaic virus	BMV
Cowpea chlorotic mottle virus	CCMV
Coat Protein	CP
Cowpea mosaic virus	CPMV
Computed Tomography	CT
Dimethylamine borane	DMAB
1-Ethyl-3-(3-dimethylaminopropyl)carbodiimide	EDC
Gold-binding peptide	GBP
Hydrogen Evolution Reaction	HER
Lateral Flow Immunoassay	LFIA
Localized surface plasmon resonance	LSPR
Metal binding peptide	MBP
N-Hydroxysuccinimide	NHS
Nanoparticle	NP
Nickel-Nitrilotriacetic Acid	Ni-NTA
Poly(allylamine) Hydrochloride	PAH
Poly-L-lysine	PLL
Photothermal Therapy	PTT
Red clover necrotic mosaic virus	RCNMV
Surface-enhanced Raman spectroscopy	SERS
Titanium binding peptide	TBP
Tomato bushy stunt virus	TBSV
Tetraethylene glycol	TEG
Transmission electron microscopy	TEM
Tobacco mosaic virus	TMV
Turnip yellow mosaic virus	TYMV
Virus-like Particle	VLP

## 1 Introduction

**Fig. 1:**
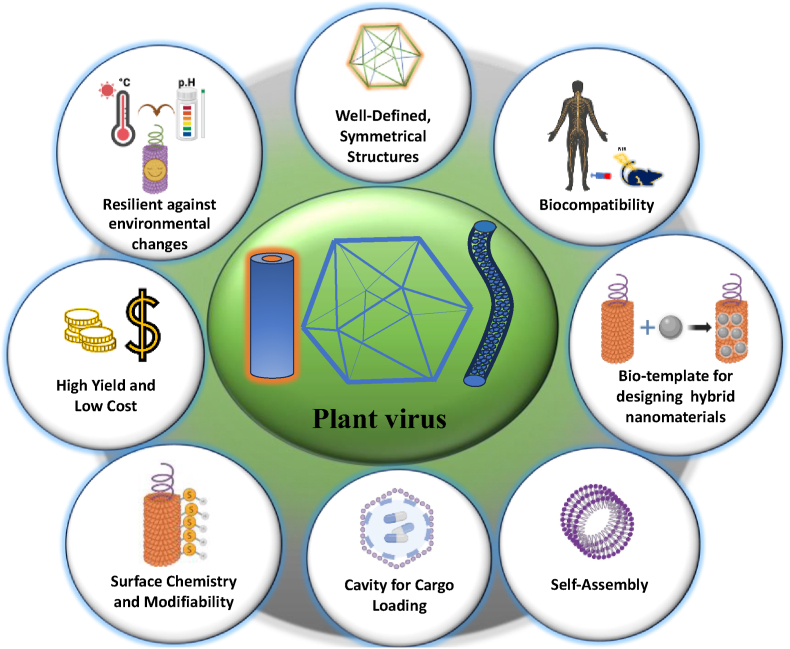
Plant viruses and their unique properties for development of nanohybrids.

Recent advances in nanotechnology have underlined the unique opportunities that plant virus capsids present as scaffolds to organize three-dimensional nanostructures for developing novel functional nanomaterials (Fig. 1). Two main components are typically found in plant viruses: genetic material (either RNA or DNA) and a capsid, which protects the genetic material. The capsid is composed by self-assembled proteins called coat proteins (CP). Two basic patterns are possible for the capsid: helical and icosahedral symmetry. For icosahedral structure, the triangulation number (T) is used by virologists to describe their size and complexity. It defines how many protein subunits are used to build each triangular face of the icosahedron. In a very simple way, the number of subunits is equal to 60xT. For example, a T = 1 virus has 60 identical subunits, while a T = 3 virus has 180. Distinct surface charges and functional groups are presented by the amino acids on their surface, which play a pivotal role in the conjugation of nanoparticles. These robust virus scaffolds, against thermal and chemical stresses, are considered safe for human interaction [[1], [2], [3]] and can be modified with a variety of functional materials to enhance their utility. Additionally, attention has always been drawn to AuNPs due to their unique properties, such as localized surface plasmon resonance (LSPR). This property is instrumental for applications involving surface-enhanced Raman spectroscopy (SERS) [4]. Their low cytotoxicity and efficiency as photothermal [5] and bio-imagining agents [6] further underscore their suitability for biomedical applications, especially in the treatment of diseases like cancer [7].

**Fig. 2:**
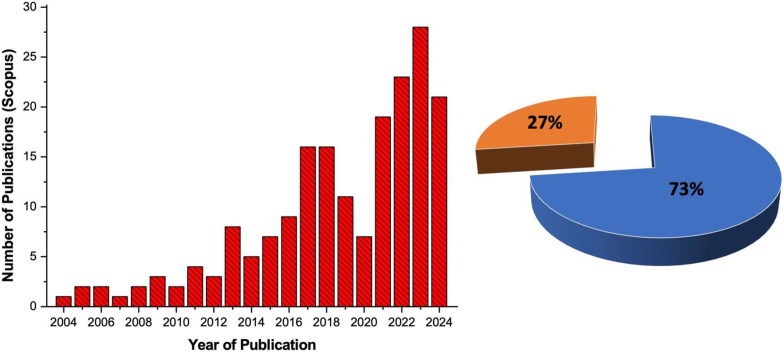
**(a)** Number of research articles in the Scopus database (using keyword gold nanoparticles and plant viruses, 2004 to 2024) on gold nanoparticles and plant viruses **(b)** Gold nanoparticles for detection of plant viruses (blue) and gold nanoparticles and plant viruses as nanohybrids (brown). (For interpretation of the references to color in this figure legend, the reader is referred to the Web version of this article.)

Over the years, review papers have been written on the study of metal nanoparticles (NPs) and plant viruses (Fig. 2) and very few (27 %) have focused on the grafting of these NPs onto plant virus capsids for nanohybrid formation. The other part (73 %) is focusing on plant virus detection using plasmonic properties of gold nanoparticles (Fig. 2b). They have been detected and treated by plasmonic nanoparticles [8], including sugarcane mosaic viruses [9] and Potato virus X (PVX) [10], with amplification lateral flow immunoassay (LFIA) techniques being employed to significantly enhance the signal intensity for early detection of viruses in plants. E.g the effectiveness of Raman spectroscopy as a high-throughput method for detecting plants infected with Turnip Yellow Mosaic Virus (TYMV), which can be distinguished from healthy, non-infected ones, was explored by Kim et al., and its potential as an alternative screening tool in agriculture was suggested [11].

Despite these reviews there has not been an effective one focusing on the application part of the plasmonic nanoparticles combined with the viral capsids recently. The integration of these two nanotools i.e viral biomolecular templates with AuNPs, opens new avenues in fields like biochemical sensing, drug delivery, catalysis, and for the other bio-electronic devices [[12], [13], [14], [15]]. The efficacy and stability of these virus-based metallic nanohybrids are contingent upon the interplay between the virus and the nanoparticles, particularly their shape, size, assembly and composition. By combining the natural precision and self-assembly capabilities of viruses with the unique optical, electronic, and chemical properties of plasmonic nanoparticles, researchers are developing innovative materials for applications in medicine, sensing, and catalysis. New ways to create highly organized nanostructures with enhanced functionality are offered by these virus-AuNP hybrids, making them a promising platform. This review focuses on detailed insights into the synthesis of plant virus-based gold nanohybrids, highlighting diverse grafting methods and addressing critical aspects of stabilization and filtration. The influence of genetic mutations in CPs on the nucleation and grafting of nanoparticles is thoroughly examined. Additionally, the morphological impact of the shape and composition of AuNPs on their applications is also discussed in detail.

## 2 Methods of grafting

**Fig. 3:**
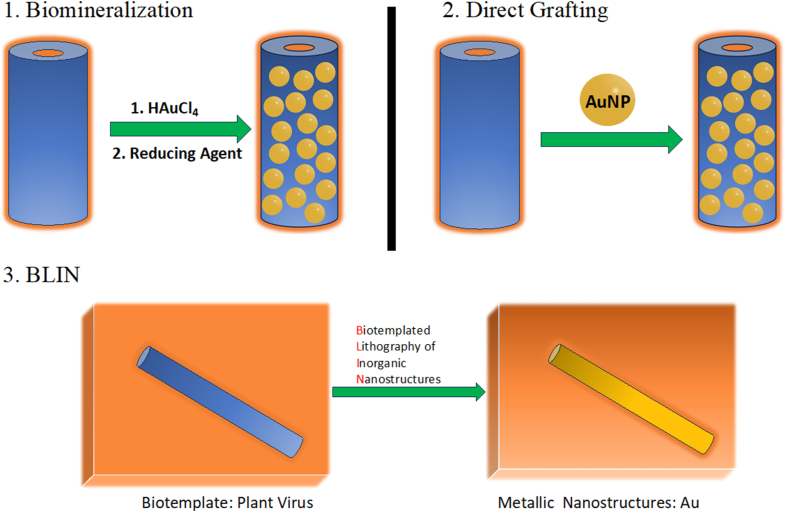
Various methods for grafting gold onto the capsid. (For interpretation of the references to color in this figure legend, the reader is referred to the Web version of this article.)

Based on the mode of grafting, the attachment of gold nanoparticles to virus capsids can be categorized into three primary methodologies (Fig. 3): (1) Biomineralization (2) Direct Grafting (3) Grafting by lithography, distinguished by its underlying mechanisms and outcomes.

### 2.1 Biomineralization

Biomineralization involves the incubation of gold salts with the capsid at a specific pH, facilitating interactions between the salt and the capsid. The reduction of the metal ions from Au^3+^ to Au^0^ is achieved through the use of reducing agents such as sodium borohydride (NaBH_4_) [16,17], hydrazine [18,19], or exposure to radiation like ultraviolet light [20], or electron beams [21] and by the self-reducing properties of amino acids like tyrosine [22]. Different reducing agents like dimethylamine borane (DMAB) [23,24] and NaBH_4_ [16] are selected for their ability to control gold ion reduction into nanoparticles, considering their effects on biological templates, nanoparticle characteristics, and safety/environmental factors.

This method results in the initial formation of small nanoparticle seeds on the virus surface, which can then be enlarged in diameter through a cyclic growth process by adding gold precursors and reducing agents [21]. Although it has been observed that the biomineralization method produces fewer free nanoparticles, the absorption of the plasmonic peak however tends to be lower due to the relatively low concentration of gold [25]. The nucleation site is also influenced by the type and position of the amino acid on the capsid [26]. To boost the colloidal stability of the nanohybrid, many coating agents like poly-L-lysine [16], and dextran [21] have been used without disturbing the actual morphology of the virus. The choice of coating depends on the desired characteristics of the nanohybrid and the specific outcomes required for each application. The hybrid is stabilized by poly-L-lysine in environments where strong electrostatic interactions are required, such as in aqueous or slightly acidic solutions. Dextran is ideal for *in vivo* applications. Being a natural polysaccharide, dextran enhances biocompatibility.

### 2.2 Direct grafting of AuNPs

Direct Grafting of AuNPs is a method where pre-synthesized gold nanoparticles are attached to the virus through covalent bonding or by strong electrostatic interaction. This can be achieved either through genetic modification of the virus capsid such as insertion of cysteine [[27], [28], [29]], histidine [20] or by the chemical reactions, such as those involving EDC (1-Ethyl-3-(3 dimethylaminopropyl)carbodiimide) and NHS (N-Hydroxysuccinimide) [30]. Subsequent separation of the free gold nanoparticles from those that are grafted onto the virus can be accomplished using techniques like ultrafiltration [31] and gel electrophoresis [30]. This method allows for the precise control over the placement and high density of gold nanoparticles on the viral capsid.

### 2.3 Lithography-Based Grafting

Lithography-Based Grafting also known as biotemplated lithography of inorganic nanostructures (BLIN) leverages the inherent structures of the virus capsids as templates for the precise arrangement of nanomaterials, thereby facilitating the creation of complex and highly ordered nanostructures. In essence, biological templates are employed by this approach to pattern and fabricate ordered inorganic nanostructures with nanoscale precision. Its strengths lie in structural accuracy and pattern reproducibility, but it often requires complex fabrication steps, including electron beam exposure or chemical etching, which may compromise the integrity of biological templates. DNA origami and TMV were used by Piskunen et al. as templates for patterning nanoscale materials onto substrates such as silicon wafers and indium tin oxide-coated glass. The approach has been used to fabricate various nanostructures, such as metallic Au, Ag, Al, and Ti, as well as semiconducting Ge nanoparticles. The technique holds promise for applications in plasmonics, biosensing, and the development of functional metamaterial [32].

Each of these methods has its specific advantages and limitations (Table 1), as their application is determined by the desired properties of resultant nanohybrid structures. The first two methods are in solution media, hence, chemical deposition which is more favorable for biological structures due to their electroneutrality. Biomineralization can also offer more size tunabilty while direct grafting has strong plasmonic signal. On the other hand, precise nanopatterns for advanced optical applications are created by lithography; however, complex fabrication is required, and yield issues can arise due to the need to preserve the integrity of biological structures. These structures are often exposed to physical and chemical stresses, such as electron beams or harsh chemicals, during patterning.

**Table 1:** Advantages, disadvantages, and stability of different methods for AuNP–plant virus nanohybrid synthesis.

Method	Advantages	Disadvantages	Stability
**Biomineralization**	- Gentle on biological templates. - Size tunability of nanoparticles. - Fewer free nanoparticles produced.	- Often low plasmonic peak absorption - Requires more time in the case of self-reduction.	Good stability, especially with stabilizers like poly-L-lysine and dextran.
**Direct Grafting**	- High density and control over nanoparticle placement. - Covalent bonding offers strong attachment.	- Requires genetic modification or chemical reactions. - Removal of free nanoparticles can be labour-intensive.	High stability with correct bonding conditions (e.g., EDC/NHS coupling).
**Biotemplated Lithography (BLIN)**	- High precision in nanopatterning. - Useful for plasmonics and biosensing applications.	- Complex fabrication involving physical and chemical stresses. - Limited stability of biological templates	Stability may be compromised during electron beam or chemical etching.

## 3 Grafting of AuNPs over different types of plant viruses

Building upon the different grafting techniques, the stability, uniformity, and functionality of gold nanoparticles are determined by the specific morphology of various plant viruses, which can be further optimized according to the virus type (Table 2) and the desired nanohybrid characteristics. Among the diverse shapes of viruses, rod-shaped and icosahedral viruses have been extensively studied for the synthesis of plasmonic-based nanohybrids, which may be attributed to their geometric simplicity and the ease of extraction from plant hosts.

**Table 2:** Virus Types, shapes, and attachment mechanisms.

Virus Type	Shape	Attachment mechanism
Tobacco mosaic virus (TMV) [16,18,20,24,33]	- Rod-shaped, helical symmetry - ∼300 nm in length, ∼18 nm diameter, with a 4 nm central channel. − 2130 identical CPs	- Electrostatic interaction (e.g., arginine residues with negatively charged citrate-coated AuNPs). - Covalent bonding (e.g., thiol-gold interactions via cysteine residues).
Tomato mosaic virus (ToMV) [34]	- Rod-shaped, hélican symmetry - 300 nm length, 18 nm outer diameter, 4 nm channel - 2130 identical CPs	- Electrostatic interactions and template mineralization for metal deposition.
BSMV [35]	- Rod-shaped - 110-150 nm in length, 20 nm in diameter - 26 CPs subunits per turn	- Electrostatic and covalent bonds together cause gold mineralization on the virus.
Cowpea mosaic virus (CPMV) [29,[36], [37], [38]]	- Icosahedral, pseudo triangulation number (T) = 3 - Outer ∼28 nm in diameter inner 20 nm - 60 asymmetric units (a 24 kD and a 41 kD subunit)	- Biomineralization for controlled nanoparticle size. - Surface modification (e.g., cationic polyelectrolyte to enhance electrostatic adsorption).
Turnip yellow mosaic virus (TYMV) [11,30,39]	- Icosahedral, (T = 3) - ∼28 nm diameter, inner 21 nm with a highly regular surface topology - 180 identical CPs	- Covalent bonding via amide or thiol groups. - EDC/NHS coupling reactions for nanoparticle attachment.
Brome mosaic virus (BMV) [[40], [41], [42], [43]]	- Icosahedral, (T = 3) - ∼28 nm diameter, inner diameter 18 nm - 180 identical CPs.	- Electrostatic interaction with negatively charged nanoparticles. - Surface modification using surfactants or specific chemical groups.
Cowpea chlorotic mottle virus (CCMV) [22,[44], [45], [46]]	- Icosahedral, (T = 3) - ∼28 nm diameter inner 18 nm - 180 identical CPs forming a porous shell.	- Encapsulation of AuNPs via charge interaction. - Mutations for enhanced binding and metallization.
Red clover necrotic mosaic virus (RCNMV) [47]	- Icosahedral, (T = 3) - Diameter ∼35 nm inner 17 nm - 180 identical CPs	- Used in encapsulating gold nanoparticles with RNA, enabling virus-like particle assembly.
Tomato bushy stunt virus (TBSV) [48]	- Icosahedral, (T = 3) - Uniform size (∼35 nm). Inner diameter 22 nm - 180 subunits.	- Histidine-Nickel (Ni-NTA) interaction.Ni-NTA nanogold binds to His-tagged proteins.

**Fig. 4:**
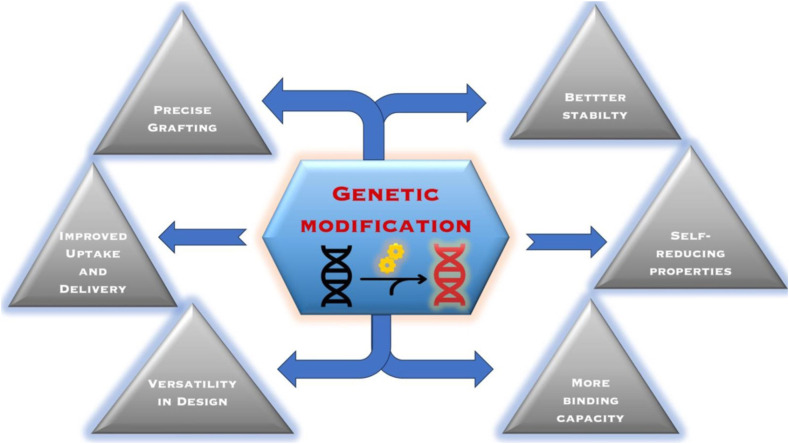
Advantages of the genetically modified plant virus capsids.

To enhance NP grafting efficiency and symmetry directed synthesis of NPs, genetic modifications are employed to modify the metal-binding amino acid composition of the virus (Fig. 4). The advantages and chemical role of residues inserted in these mutations are explained in Table 3. Genetic mutations of plant viruses grant high selectivity and facilitate the development of numerous tools for extensive analyses. Typically, modifications to the protein shell are achieved by appending peptides of 2–20 amino acids or substituting an amino acid with its corresponding residue. Mutations introducing different amino acids such as cysteine [25] or lysine [28], onto these virus capsids have facilitated advancements in surface chemistry and functionalization.

**Table 3:** Mutations in virus capsids and their advantages for gold nanoparticle grafting.

Mutation	Virus	Advantage for Grafting Nanoparticles
Cysteine [23,[27], [28], [29],^35^,^51^]	TMV CPMV	- Enhanced AuNP binding via thiol-gold interactions; improved stability and precise control over nucleation sites. - T158C → Outer surface [25], TMV1Cys → Outer surface [23,51], Inserted at N-terminus → Outer surface [24], Cysteine (T103C) arrayed → *inner surface* [26]
Lysine [28]	TMV	- The introduced lysine residues enable efficient attachment of gold-binding peptides (GBP) through covalent bonding with the linker. - Lysine, with its amine (-NH_2_) group, can also enhances electrostatic and covalent interactions for nanoparticle attachment - T158K → Outer surface
Histidine [19,20,22]	TMV CPMV	- Enhances self-assembly and bonding through imidazole functionalities, allowing control over nanoparticle placement. - N or C-terminal → Outer surface [19], C-terminal → Outer surface [20,22],
Tyrosine [22]	CCMV	- Enables self-reducing properties for *in-situ* reduction of metal ions, eliminating the need for external reducing agents. - Tyrosine provides a phenolic –OH group capable of reducing metal ions like Au^3+^ to Au^0^, supporting in-situ nanoparticle synthesis. - Near the C-terminus → Outer Surface
Gold-Binding Peptide (GBP) [17]: LKAHLPPSRLPS	TMV	- Promotes selective gold nanoparticle nucleation, forming highly organized nanostructures. - GBP is genetically fused to the C-terminus of the CP → Outer surface of the virus
A8 Peptide [52]: VSGSSPDS	TMV	- Facilitates selective binding of colloidal gold nanoparticles specifically at the ends of TMV nanorods. - Genetically fused directly to the C-terminal end of TMV coat protein → Outer-surface**.**
Metal Binding Peptide (MBP) [49]: SEKLWWGASL	TMV	- Eco-friendly and controlled synthesis of stable metal nanoparticles without harmful chemical reductants. - Located in the C-terminal surface-exposed loop of the TMV coat protein → Outer-surface**.**
Titanium binding peptide (TBP) [17]: RKLPDA	TMV	- Creates smaller, more uniform gold nanoparticles for better optical materials. - Genetically fused to the C-terminus of the CP → Outer surface

Initially, wild-type (wt) capsid has facilitated the production of metallized virus structures. Although unmodified viral capsids can bind metal ions via surface-exposed hydroxyl (OH) and carboxyl (COOH) groups, as well as through OH and primary amine groups in its internal channel, these interactions do not efficiently reduce sequestered ions into metal NPs or achieve virus metallization on their own. Therefore, the approach of modifying the CP avoids the use of external reductants, taking advantage of the intrinsic reducing capabilities of surface-displayed peptides for NP production. For example the reduction of metal ions may be aided by the hydroxyl groups in tyrosine, carboxyl groups in asparagine and glutamine, and indole groups in tryptophan [22,49]. For example, certain icosahedral viruses have been genetically modified with amino acids like tyrosine [22] and cysteine [29] for the *in-situ* reduction and efficient binding of AuNPs with the capsid respectively (Table 4). Other mutants like His-tagged virus-like particles (VLPs) in synthesizing uniform gold metallic layers, used UV photoreduction method after incubation of metal salt [20]. It is proposed that these peptide sequences modify the charge balance on the capsid surface, potentially enhancing nanoparticle nucleation under reducing conditions. The absence of exogenous reducing agent can be important for the wide range of the applications by enhancing the stability and biocompatibility. A study by Bruckman et al. examined the incorporation of a His-tag into the CP, which was found to significantly enhance its self-assembly capabilities. A diverse range of nanostructures including disks, helical rods, and fibers is demonstrated by the His-tagged CP (His-TMV-CP), which are more complex and stable compared to those formed by the wild-type-CP. The addition of the His-tag enables the control of assembly through interactions that can be tuned by modifying environmental conditions such as pH, ethanol presence, and nickel-nitrilotriacetic acid (Ni-NTA) concentration. This versatility makes His-TMV-CP a promising scaffold for the development of advanced nanostructured materials [50].

**Table 4:** Comparison of TMV-wt and TMV-T158C. Growth rate with increasing electron dose rate, size nanoparticles and association constant of AuCl_4_^−^ with Virus.

Virus Type	Growth rate (%/s)			Size of AuNPs (nm)	logK_a_
	e^−^ dose rate (e^−^/Å^2^·s)				
	0.14.	0.55	2.2		
TMV-wt [21]	1.12 ± 0.10	1.75 ± 0.16	3.53 ± 0.32	5.8 ± 1.2	4.80 ± 0.05
TMV-T158C [25]	1.28 ± 0.07	2.59 ± 0.08	9.1 ± 1.0	12.4 ± 2.8	5.95 ± 0.15

### 3.1 Rod shaped viruses

#### 3.1.1 Tobacco mosaic virus (TMV)

**Fig. 5:**
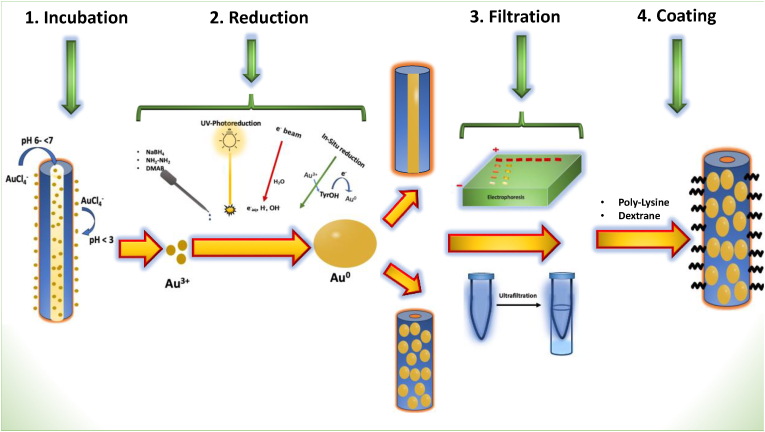
General scheme of forming Plant Virus-AuNP Nanohybrid (TMV).

Viral capsids, including the widely studied rod-shaped virus TMV, have been distinctively utilized for the synthesis of gold nanoparticles grafted onto their capsid [16] (Fig. 5). Besides its robust nature, the extensive utilization of TMV can be attributed to its early identification and comprehensive analysis of its structure and composition. TMV exhibits a 300 nm length and a cylindrical shape, with a 4 nm hollow cavity, which facilitates novel developments in leveraging rod-shaped viruses for wide range of applications through the conjugation of its capsid with AuNPs. Given TMV's non-pathogenic nature to mammals and its biodegradability, it is considered a viable candidate for various biomedical applications [53].

By manipulating the pH of the surrounding medium, the nucleation site can be determined, in hollow viruses such as TMV, where the charge disparity between the interior cavity and outer surface can be pH-regulated [33]. The resulting interactions between the virus and nanoparticles predominantly include electrostatic and covalent bonds, depending upon the functional groups present on the amino acids. At low pH, according to the isoelectric point of TMV (between 3.5 and 4.6) the outer surface of TMV is prone to electrostatic interactions with negatively charged citrate-coated gold nanoparticles [54].

The synthesis of AuNPs by mineralization, utilizing TMV as a biological template, was first carried out by Dujardin et al., in which gold salts were incubated with TMV viral solution at low pH and subsequently reduced using hydrazine [18]. Following reduction, transmission electron microscopy (TEM) images revealed the presence of small AuNP seeds on the exterior of the TMV capsid. This early work laid the foundation for understanding the potential of TMV as a versatile nanomaterial template, revealing the importance of biotemplating in controlled nanoparticle synthesis**.** Brom et al. reported that enhanced size uniformity and growth of AuNPs were achieved through cyclic amplification [16]. Their use of multiple reaction cycles significantly improved the monodispersity of the gold nanoparticles, providing a promising route for producing highly uniform metallic nanoparticles on biological templates, with potential for large-scale applications. Royston et al. developed a silica coating strategy that promotes not only the stability of the biotemplate but also its affinity for metal ions [55]. By integrating silica as a protective and functional layer, this method effectively stabilized the TMV template under metal deposition conditions and enhanced the reproducibility of the synthesis process, addressing the stability concerns. Zhou et al. developed a one-dimensional (1D) nanohybrid, in which AuNPs were surface-deposited through rod-like TMV self-assembly and mussel-inspired dopamine polymerization with in situ gold ion reduction; however, an extended period was required for the formation of the AuNP hybrid. This approach not only demonstrated a novel method for 1D hybrid nanofiber fabrication, but also highlighted the use of TMV as a template for advanced nanostructured materials that could be utilized in endocytic pathway sensing and drug delivery applications [56]. Recently, it was demonstrated by Pussepitiyalage et al. (2024) that moderate heating in an aqueous solution promotes the electroless deposition (mineralization) of noble metals (Au, Pd, Pt) directly onto the mutant TMV CP, forming metal-coated rod-like structures. The heat accelerates the reduction of metal ions by the electron-rich amino acid residues on the virus surface, enabling metals like Pd, Pt, and Au to nucleate and grow directly on the virus templates [35].

#### 3.1.2 Mutants of TMV

Beyond wild-type viruses, genetic mutations in viral coat proteins offer further control over nanoparticle binding and morphology. This section explores how specific mutations, such as those introducing cysteine or histidine residues, enhance the precision and performance of AuNP grafting. For example, covalent bonds can be formed by cysteine residues through thiol-gold (Au–S) interactions. An in-depth study for the nanoparticles nucleation rate was recently done by measuring the gold nanoparticle growth rates on a cysteine-modified tobacco mosaic virus (TMV T158C) on the outer-surface of CP by the liquid *in-situ* TEM and compared the growth rate to the TMV-wt at different electron dose rates (Table 4) [21,25]. Here electron beams generated water reactive species that initiated nucleation without using any chemical reducing agent. The TMV-T158C had higher gold growth rate and the size of the AuNPs formed after cyclic growth method was significantly higher as well. The role of cysteine in modifying TMV nucleation rates was collectively confirmed by these methods, and its contribution to enhancing the Surface-Enhanced Raman Spectroscopy (SERS) performance of grafted AuNP assemblies on TMV-C scaffolds was also highlighted in this study. It demonstrated that faster nanoparticle growth, higher NP density (NP/nm^2^), and optimal particle size significantly improve signal enhancement for biosensing applications, which will be discussed later in the application section 6.1.

Metal cluster deposition on a genetically engineered TMV was improved by Lee et al. through the insertion of two cysteine residues into the amino terminus of its coat protein, which enhanced metal binding and enabled more stable and denser coatings of gold, silver, and palladium clusters compared to the unmodified virus [24]. The process was further refined by Lim et al. through quantitative analysis of the biosorption of Au(III) and Pd(II) ions on TMV1Cys, investigation of the effects of ion concentration and temperature on metal uptake, and optimization of metal deposition by adjusting these factors [23].

After enhancing metal binding through cysteine-based modifications***,*** the introduction of a His-tag further refined the process, offering improved control over nanoparticle nucleation and growth, particularly for gold nanocrystals. It was detailed in the study of Liu et al. that the genetic modification of TMV CP, through the addition of a His-tag, modifies the assembled structures to create a more favorable one-dimensional template. This modification facilitates the nucleation and growth of gold nanocrystals on the template, enhancing both the structural integrity and the density of Au-nanocrystals. This study illustrates that genetic alterations can significantly influence the structural configuration of TMV CP-based nanocomposites, expanding their application range and improving their suitability for nano-electrical device fabrication due to enhanced metallization and plasmonic properties [19]. This approach was advanced by Wnęk et al. through the presentation of a straightforward bacterial expression system for the rapid production and purification of recombinant chimeric TMV CP with C-terminal peptide tags. These proteins retain their self-assembly capability into virus-like arrays at acidic pH levels. Utilizing a C-terminal His-tag, the team crafted virus coat protein-templated nanorods that uniformly bind gold nanoparticles. Subsequent gold atom deposition and thermal annealing transform these into gold nanowires. This process demonstrates the utility of His-tagged VLPs in synthesizing uniform gold nanowires, following incubation with citrate-stabilized gold nanoparticles and UV photoreduction [20].

A genetic fusion of material-binding peptides to the TMV CP was engineered by Kobayashi et al., specifically incorporating a gold-binding peptide (GBP; sequence: LKAHLPPSRLPS) at the C-terminus and a titanium-binding peptide (TBP; sequence: RKLPDA) on TMV's outer surface. This modification facilitates metal crystallization. When utilizing TBP-engineered TMV, nanoparticles with an average size of 5 nm were produced, showcasing a smaller standard deviation (±1.06 nm) in size compared to those generated on TMV-wt (±2.18 nm). Conversely, the application of GBP-fused TMV resulted in nanoparticles with an average diameter of 10 nm. These observations suggest that genetically altered TMVs serve as effective templates for the development of optical metamaterials, a property attributable to GBP's nucleation-enhancing ability. Once AuNP nuclei form preferentially on GBP, their rapid growth impedes the development of other nanoparticles [17]. Following this, the incorporation of a metal ion binding and reducing capacity (MBP) motif (SEKLWWGASL) into a surface-exposed region of the TMV coat protein (CP) was described by Love et al. This modification resulted in the formation of a thin, networked structure markedly different from the typical TMV-wt rod morphology. This genetically engineered TMV variant demonstrates ability to the formation of discrete gold nanoparticles ranging from 10 to 40 nm in diameter upon exposure to potassium tetrachloroaurate, without the need for exogenous reductants offering a greener, more sustainable method. This process, is slower as it takes 48 h, leverages the intrinsic reductive potential of the displayed peptide motif to synthesize nanoparticles [49].

Finally, the incorporation of MBP subunits as C-terminal extensions into TMV nanorods synthesized using pEff-based expression was demonstrated by Saunders et al. The binding of colloidal gold was observed at both ends of the Coat Protein/Origin of Assembly Sequence (CP/OAS)-A8 nanorods, where the A8 amino acid (A8 peptide–VSGSSPDS) extensions are more prominently displayed compared to the surface of the nanorod cylinder [52]. The further potential of GBP on TMV for enhanced biomineralization processes and the formation of gold nanowires has been explored by Ma et al., providing a more eco-friendly and scalable approach. He introduced a method for synthesizing gold-nanoparticle-coated TMV in vitro or in plant extracts, employing a TMV variant displaying a cysteine-terminated GBP suitable for gold biomineralization. This approach advances the development of engineered living materials, proposing the in-situ formation of gold nanowires within plant tissues for applications in sensing and energy harvesting [28].

Each study represents a progression in modifying TMV for metal nanoparticle synthesis, starting from single mutation to the introduction of metal-binding peptides to enhancing stability and facilitating in-situ formation of gold nanoparticles.

#### 3.1.3 Nanorings/nanodisks of genetically modified TMV

The RNA-free viral coat protein TMV CP has been engineered to adopt a ring-like conformation by altering pH or ionic strength of the medium [57], temperature [58] or by self-assembly of amino acids [59], leading to the formation of disk like nanohybrids. The use of TMV nanorings/nanodisks instead of full rod-shaped TMV structures in conjunction with AuNPs can provide several specific advantages due to differences in geometry, surface area, and structural properties. While rod-shaped TMV structures offer their own benefits, nanorings/nanodisks provide can provide better performance in specific applications for example nanorings can offer a higher surface-to-volume ratio for catalytic applications compared to rods of the same material.

The successful creation of three-dimensional, solution-stable, sub-23 nm gold nanoparticle rings using a S123C mutant TMV CP as a template was reported by Zahr et al. (2012). The assembled TMV CP disks selectively bound bis(p-sulfonatophenyl) phenylphosphine BSPP-passivated gold nanoparticles through electrostatic interactions, resulting in metallic rings [60]. This approach marked a significant step in the development of nanoparticle ring formation using TMV, establishing the foundation for future improvements in nanoparticle organization and stability Zhang et al. (2019) developed a sophisticated method by which organized nanostructures, ranging from zero-dimensional to two-dimensional arrays using functional nanoparticles, were constructed. This was achieved by genetically modifying a TMV disk. By mutating a cysteine residue at the 103rd position to introduce a thiol ligand at the disk's core, and binding differently sized gold nanoparticles (AuNPs) selectively, they crafted unique TMV-AuNP formations. The disk's outer edge was further modified with histidine residues to provide imidazole functionalities, enabling the formation of an AuNP ring at the periphery and a solitary AuNP at the center in different instances. The histidine's chelating ability with divalent metals led to the construction of two-dimensional TMV layers with honeycomb patterns, over which various AuNP arrays were engineered by adjusting the AuNP-imidazole interaction. Additionally, this TMV framework with dual functional groups guided the creation of binary nanoparticle lattices, comprising quantum dots and AuNPs, demonstrating a versatile platform for nanoparticle assembly [27]. This work set the stage for subsequent developments, as Zhang and colleagues later demonstrated a method for creating orderly one-dimensional (1D) nanostructures, such as nanochains and nanowires, by employing the TMV disk as a nano-scale transport mechanism. This process involved utilizing the thiol ligands that were engineered to be present on the interior surface of the TMV disk to attract different types of NPs, including AuNPs and silver sulfide quantum dots (Ag_2_S QDs). These particles were incorporated into the TMV disk's cavity, creating a TMV-NP complex. Furthermore, the encapsulation within the TMV capsids allowed these NPs to serve as nucleation points, promoting their directional extension into nanowires via a process known as *in-situ* deposition. This innovative approach leverages the restrictive environment provided by the TMV capsids to control NP growth, which is a key step in the synthesis of advanced nanomaterials [31]. The potential of the TMV scaffold was expanded by Petrescu et al. by selecting the TMV coat protein for its natural ability to self-assemble into highly symmetric, planar disks that serve as precise scaffolds for organizing nanoscale components, particularly in ring shapes. Mutations were introduced in the coat protein to present solvent-exposed primary amines along the periphery, enabling site-selective functionalization with AuNP-binding ligand (lipoic acid via NHS ester chemistry to allow gold−thiol bond, producing symmetrical plasmonic nanorings) [59].

#### 3.1.4 Barley stripe mosaic virus (BSMV)

In the study of Pussepitiyalage et al. BSMV serves as a sustainable biotemplate for the mineralization of noble metals like palladium (Pd), platinum (Pt), and gold (Au) by incubating in aqueous metal precursor solutions. The mineralization process occurs through both covalent and electrostatic interactions between metal precursor ions and the CPs, allowing efficient adsorption and reduction of metal ions on the virus surface without external reducing agents or harsh conditions. Compared to TMV, BSMV exhibits faster adsorption kinetics and thicker metal coatings due to its higher isoelectric point, enabling more positively charged sites to bind metal ions. This makes BSMV a promising biotemplate for producing metal nanorods applicable in catalysis, electronics, and sensing [35].

### 3.2 Icosahedral viruses

Similar to rod-shaped viruses, both wild-type and mutant icosahedral viruses are functionalized with AuNPs. Various icosahedral-shaped viruses serve as bio-templates for nanohybrid construction, with their selection depending on the capsid composition, which influences the stability of the resulting nanohybrid. The diversity of the type of virus attached with plasmonic NPs is more than the rod-shaped virus Table 2.

#### 3.2.1 Cowpea mosaic virus (CPMV)

In an earlier study, a cationic polyelectrolyte, poly(allylamine) hydrochloride (PAH), was utilized by Aljabali et al. to modify the surface of CPMV for the templated synthesis of narrowly dispersed gold nanoparticles. This modification facilitates the electrostatic adsorption of anionic gold complexes, which are subsequently reduced under mild conditions to form a metallic gold coating. The resulting CPMV-polyelectrolyte conjugate, CPMV-PA, exhibits an increased surface positive charge compared to the wild-type, enhancing its ability to attract anionic gold complexes. The gold-coated surface of CPMV-PA can be further modified using thiol reagents, including thiolated-oligosaccharides or cysteamine. Furthermore, the interaction of CPMV-PA with preformed gold nanoparticles leads to the self-assembly of large, hexagonally packed, tessellated spheres, showcasing a novel method for creating complex nanostructures [37].

Directed self-assembly techniques were employed by Lebedev et al. to accurately position AuNPs with diameters of 24–30 nm on VLPs, creating nanoclusters (NCs) and comparing them with their genetically engineered version of the CPMV (BC-NC) analogues. They demonstrated that both VLP-NCs and BC-NCs are robust biophotonic complexes capable of local electromagnetic field concentration and providing spatially-resolved Raman enhancement. This enhancement is crucial for the detection and identification of both the virus coat protein and surrounding molecules, such as DNA. The VLPs used resemble the wild-type CPMV in structure but are devoid of genetic material (RNA) within the capsid, making them ideal for sensing applications due to their material composition and the absence of nucleic acids. To functionalize the VLPs, protected-artificial thiols (SHR) were incorporated using the linker N-Succinimidyl S-acetylthiopropionate, leveraging the VLP's similarity to CPMV-wt and its proven efficacy in materials applications [38].

A self-assembly approach to construct three-dimensional, icosahedral plasmonic nanoclusters was developed by Fontana et al. These clusters were composed of genetically modified cowpea mosaic virus (BC-CPMV) entities with engineered CPMV to present cysteines groups (SH) at the BC-loop for a total of 60 thiols per capsid, each bearing 6–12 gold nanospheres (Au NSs) covalently attached at locations predetermined to achieve icosahedral symmetry. The diameters of these Au NSs were comparable to that of the CPMV itself. The team analyzed the bulk absorbance of these nanoclusters in aqueous suspension, successfully mirroring the major spectral features through three-dimensional finite-element simulations [29]. This type of site-specific mutation, introducing thiol groups at geometrically strategic points, provides a powerful strategy to enforce symmetric nanoparticle arrangement crucial for tailoring optical and plasmonic properties.

#### 3.2.2 Turnip yellow mosaic virus (TYMV)

In the study of Nguyen et al., TYMV particles were used to structure gold nanoparticles in 3D through two new covalent bonding methods. The first involved forming an amide bond between the virus capsid and functionnalized AuNPs with amine groups, while the second modified the TYMV capsid to add thiol groups. SERS experiments on these grafted AuNPs enhanced the Raman band intensity of a probe molecule, BPE [39]. A few years later, the same attachment mechanism was utilized, and TYMV was used as a platform for the novel assembly of AuNPs on its outer surface. The nanoparticles, coated with 6-amino-hexanethiol (AHT) for AuNPs, were attached to TYMV using an EDC/NHS coupling reaction. These TYMV-based nano-biohybrids showed solubility in biological media and exhibited superior photothermal effects compared to the unbound nanoparticles, showcasing their potential in biomedical applications [30].

#### 3.2.3 Cowpea chlorotic mottle virus (CCMV)

The synthesis of gold nanoparticles using the metal precursors HAuCl_4_ and AuClP(CH_3_)_3_ in conjunction with various viral capsids was investigated by *Slocik* et al. They worked with the empty capsid of SubE expressed in yeast, an engineered virus with HRE peptides (HRE sequence AHHAHHAAD) inside its cavity (HRE-SubE), and the wild-type CCMV virus particles. The synthesis occurred through two distinct pathways: firstly, the viral capsid's surface tyrosine residues facilitated the reduction of AuCl_4_^−^, and secondly, the reduction of the virus loaded with the gold(I) precursor AuClP(CH_3_)_3_ using borohydride. This biomimetic approach underscores the potential of tyrosine residues within peptide binding domains of viral cages or protein tranplates to reduce gold. Additionally, the study utilized the virus's unique surface topology, symmetry, and intrinsic histidine binding/nucleation sites in combination with the precursor AuClP(CH_3_)_3_, which selectively binds to the virus, to template the synthesis of nearly monodisperse zerovalent gold (Au^0^) nanoparticles [22].

## 4 AuNP as template for directing self-assembly of CPs

**Fig. 6:**
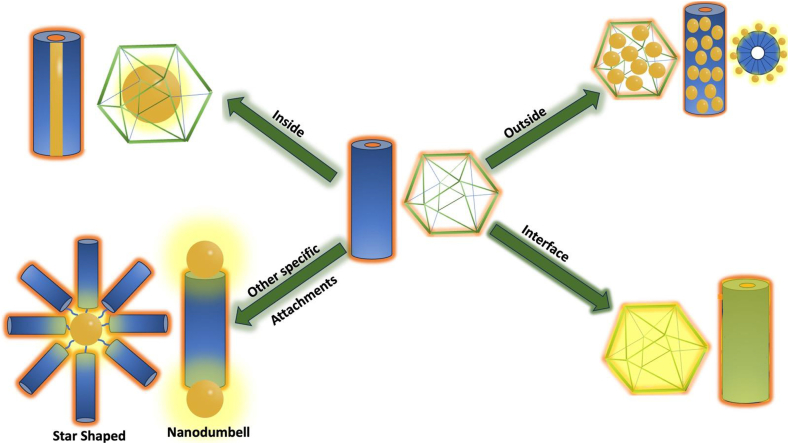
Various attachment sites of gold nanoparticles on the plant viral capsid. (For interpretation of the references to color in this figure legend, the reader is referred to the Web version of this article.)

While virus capsid have been used as templates for directing the growth of nanoparticles the reverse has also been achieved. Indeed a metal core can direct the assembly of capsid to form different shapes and sizes of VLPs. (Fig. 6). AuNPs serve as excellent templates for capsid self-assembly, providing precise size and shape control, enhanced stability, and high encapsulation efficiency. Their functionalization and tunable optical properties make them ideal for applications in drug delivery, biomedical imaging, and the development of plasmonic nanomaterials.

The virus used most frequently in these studies is the icosahedral viruses over rod-shaped viruses**.** It stems from their size and geometry which is optimal, with a diameter that suits a range of nanoparticle sizes, allowing for efficient encapsulation without compromising structural integrity. Icosahedral capsids offer size flexibility based on their triangulation number (e.g., T = 1, T = 3), making them more versatile for nanoparticle encapsulation compared to the rigid structure of rod-shaped capsids. Icosahedral capsids assemble through cooperative interactions, often requiring fewer steps and conditions than rod-shaped capsids, which can be more complex due to their elongated structure. Key parameters contributing to effective encapsulation include high charge density, specific pH conditions, ionic strength, and surface coating of nanoparticles like carboxylate-terminated thiolalkylated coatings. Additionally, core size and ζ-potential also play essential roles in controlling encapsulation efficiency and structural integrity.

### 4.1 Brome mosaic virus (BMV)

The pioneering use of Rayleigh resonance (RR) spectroscopy on single BMV capsids, measuring 28 nm in diameter and containing internalized gold nanoparticles ranging from 2.5 to 4.5 nm, was reported by Dragnea et al. They innovated a method to partially substitute the naturally occurring negatively charged RNA within the virus with these gold particles. This was accomplished through the association of negatively charged, citrate-stabilized gold nanoparticles and the basic residues inside the internal space of the BMV capsids [40]. Building on this pioneering substitution strategy, BMV CPs were successfully reassembled by *Chen* et al. around negatively charged AuNP cores in vitro, forming VLPs. They investigated the optical properties and the effects of core size and coating on these particles. The findings show that a closed shell forms around the core, which can be adjusted using different nanoparticle coatings. This work is suggestive of potential applications in real-time viral tracking and chemical sensing, as well as of the possibility of maintaining some native virus coat functions [61]. In continuation of the previous year's work, the development of VLPs using a BMV protein coat around a functionalized AuNP core was described by Chen et al. The study showcases a successful example in which the electrostatic properties of viral nucleic acids are mimicked by AuNPs to initiate the assembly of VLPs. These particles, which undergo pH-induced swelling similar to natural viruses, achieve a high encapsulation yield of over 95 %. Through transmission electron microscopy and dynamic light scattering, the VLPs appear homogeneous and are produced without unwanted aggregates or empty capsids. This achievement contrasts with other studies of lower yield attempts using citrate or streptavidin-stabilized gold particles, which suffered from issues like nanoparticle aggregation and competitive empty capsid formation. A novel method employing carboxylate-terminated thiolalkylated tetraethylene glycol (TEG)-coated particles has been proven effective, with stability enhanced and unwanted interactions reduced, thereby facilitating the self-assembly of VLPs. This advancement enables the creation of functional probes that could be utilized for enhanced biomedical imaging and sensing applications [62].

Insights into structure–function relationships laid the groundwork for further exploration of virus assembly mechanics, as demonstrated in the work of Sun et al., in which the self-assembly of VLPs composed of an icosahedral virus protein coat encapsulating functionalized spherical nanoparticle cores was explored. Using electron microscopy, researchers elucidated the structures of VLPs formed from BMV CPs and AuNPs. Varying the gold core diameter allows control over the capsid structure and packaging efficiency. These VLPs mimic three classes of viral particles found in cells (T = 1, 2, and 3) and form three-dimensional crystals under specific conditions, exhibiting optical properties influenced by multipolar plasmonic coupling. This system demonstrates the potential for regulated nanoparticle self-assembly and plasmonic metamaterial applications [42]. This technique advances previous self-assembly methods by introducing variability in the core size, enhancing the ability to fine-tune the structure and functionality of VLPs for specific applications. To further optimize assembly efficiency and material properties, the role of surface charge density was investigated by Daniel et al. using a mixture of thiolated ligands to decouple charge and size effects. It was observed that a critical charge density is required for assembly, which affects the yield of encapsulation without capsid size or structure variation. The assembly process involves forming a stabilized shell around the core at neutral pH, then rearranging proteins when pH decreases. High charge densities yield the best encapsulation efficiency, suggesting that capsid stability can be manipulated through surface chemistry for potential use in targeted delivery applications [43]. This approach improves upon previous methods by better controlling the assembly dynamics through surface chemistry, resulting in higher encapsulation yields and more stable structures.

Comprehensive protocols for such templated self-assembly were outlined by Tsvetkova et al., who described high-efficiency in vitro self-assembly strategies using functionalized gold nanoparticles within viral protein cages from BMV and other viruses. BMV has been used to create VLPs by mixing coat proteins with negatively charged nanoparticles under specific conditions. These protocols illustrate how viral protein cages can be utilized to create novel nanomaterials, leveraging the inherent size control and environmental responsiveness of viruses combined with the properties of encapsulated nanoparticles [41]. This method represents an improvement over previous protocols by offering a more streamlined approach to nanoparticle encapsulation, reducing time and increasing encapsulation efficiency. Elaborating on how external factors influence this process, Tsvetkova et al. explored the assembly pathways of BMV VLPs under varying pH and ionic conditions. They found that the assembly mechanism can shift between cooperative and non-cooperative adsorption of protein subunits onto the nanoparticle, influenced by buffer conditions. At high pH and low ionic strength, a non-cooperative Langmuir adsorption model describes the initial random protein associations. Conversely, at low pH conducive to *in vivo* virion assembly, the protein adsorption is cooperative, requiring a critical nucleus larger than that needed for forming empty capsids. This research underscores how environmental conditions can fundamentally alter the assembly pathways and efficiency of VLPs, suggesting variable assembly processes in viral systems [63]. This work expands on earlier findings by highlighting the importance of pH conditions in the VLP assembly process, offering more precise control over nanoparticle interaction.

Taking morphology control to new dimensions, the assembly of icosahedral virus coat proteins into elongated polyhedral structures using a gold nanorod template was demonstrated by Zeng et al. Using this method, spherocylindrical shells with hexagonally packed bodies and icosahedral caps were formed by the coat proteins. *In situ* atomic force microscopy revealed features such as chirality and structural defects, showing how nanorod aspect ratio and curvature mismatch guide defect generation and structural polymorphism in viral assemblies [64].

### 4.2 Cowpea chlorotic mottle virus (CCMV)

Inspired by the success of BMV-based systems, researchers turned to structurally similar viruses such as CCMV to further refine nanoparticle encapsulation strategies and explore broader applications.

In 2009, Aniagyei et al. conducted a pivotal study that explored how a mutant of CCMV, which lacks a significant portion of its N-terminal domain, can still form VLPs when assembled around rigid nanoparticle cores, rather than RNA. This mutant, forms capsids of varying sizes; however, when nanoparticles are used as the core, the size distribution narrows and resembles that of the wild-type virus. This behaviour contrasts with the broader size distribution seen with empty mutant capsids and points to potential applications in biomedicine, where controlling capsid size could enhance drug delivery effectiveness. It is suggested by the findings that the specific interactions between the CP and solid cores could be key in designing effective virus-based systems [45]. Building upon this, in 2016, Liu et al. utilized the coat protein of CCMV to encapsulate gold nanoparticles of varying sizes, using a simplified one-step method that achieves up to 99 % efficiency with certain surfactants*.* The virus-like particles formed correspond to different icosahedral triangulation numbers (T = 1, T = 2, T = 3), with the best encapsulation results seen with smaller nanoparticles, particularly those 7 nm in diameter, which fit optimally into the smallest (T = 1) CCMV capsids. Larger particles up to 17 nm can be accommodated in T = 3 structures but with varying encapsulation efficiencies. Surfactant's electrostatic properties also significantly influence the efficiency of particle formation, with a ζ-potential of −25 mV proving most effective. This research provides a fundamental approach for creating biohybrid assemblies using CCMV capsids as nanocontainers, potentially applicable across various nanotechnology and biomedicine areas [46]. This work enhances previous methods by improving the encapsulation efficiency with optimized surfactant and nanoparticle size conditions, leading to higher yields of homogenous VLPs.

### 4.3 Other viruses

Subsequent research expanded the scope of nanoparticle encapsulation by applying similar virus-like assembly strategies to other viruses such as red clover necrotic mosaic virus (RCNMV), thereby enhancing structural control, functional diversity, and efficiency in biohybrid nanomaterial design*.* A strategy to encapsulate AuNPs within a viral CP shell of RCNMV was presented by Loo et al. RNA sequences are attached to Au nanoparticles in the process, which are then recognized by the CP to trigger self-assembly, resulting in VLPs. After incubation and assembly, the VLPs are purified, revealing an average size of 33.5 nm. Approximately 35 % of the Au nanoparticles were successfully encapsulated, showing structural integrity. This method provides a controlled way to encapsulate nanoparticles or other cargo using viral CPs, offering stability and specificity [47]. In the study of Eber et al. where the encapsulation of NPs was not exactly done but they developed a procedure for RNA-directed assembly that creates bioinorganic hybrid nanostars with multiple virus-derived arms, offering a high and adjustable protein surface area to gold nanoparticle core ratio. This method combines inorganic nanoparticles with well-organized viral complexes, producing a new generation of large, ordered multicomponent nanocomposites [65]. By introducing RNA-directed assembly, this work enhances the versatility of nanoparticle functionalization, improving the adaptability and assembly precision compared to traditional methods.

The use of AuNPs as templates for viral coat protein self-assembly enables precise control over the size, shape, and functionality of VLPs, with high encapsulation efficiency and structural integrity. As shown in Fig. 6, the localization of AuNPs inside, outside, or at the interface of capsids offers diverse assembly pathways, supporting advanced applications in nanomedicine and plasmonic materials.

## 5 Grafting of viral capsids with NPs of different shapes and compositions

**Fig. 7:**
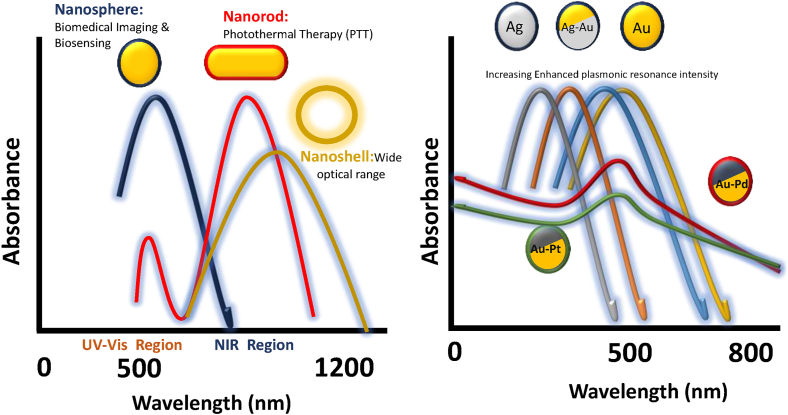
Shifting of plasmon band with shape and composition of nanoparticles.

Despite the differing morphology of viral capsids, new avenues for broader studies are also opened by the shape and composition of the grafted nanoparticles. Both composition and shape of the NPs is critical, as it can diversify the spectrum of potential applications (Table 5, Table 6). This is due to the shift in the plasmon band with varying shape and composition and also the complex geometries that contribute to efficient properties like LSPR (Fig. 7).

**Table 5:** Comparative properties and applications of gold-based alloy nanoparticles.

Alloy NP	Plasmon Bands	Applications	Advantages over AuNPs
**Gold-Silver (Au-Ag)**	Broad plasmon bands around 400–700 nm, tunable based on composition and size.	- Plasmonic sensing [66]. - Enhanced Photothermal therapy (PTT) [67]. - Surface-enhanced Raman scattering (SERS) [68]. - Catalysis for oxidation reactions [69].	- Enhanced plasmonic activity due to silver's higher electron density. - Broader tunability of optical properties.
**Gold-Platinum (Au-Pt)**	Plasmon bands less pronounced (broad absorption in 500–800 nm range).	- Fuel cell catalysts [70]. - Drug delivery and cancer treatment [71].	- Improved stability due to Pt's inert nature. - Superior catalytic activity compared to Au alone. - Higher resistance to oxidation and degradation.
**Gold-Palladium (Au-Pd)**	Plasmon band visible in the range of 500–600 nm, depending on composition.	- Highly effective in hydrogen sensing applications [72]. - Electrocatalytic applications [73].	- Smaller nanoparticle sizes and homogeneous alloying enhance sensitivity - Synergistic effects enhance catalytic activity and selectivity.
**Gold-Platinum-Palladium (Au-Pt-Pd)**	No distinct plasmon band due to strong damping; broad absorption across visible and near-infrared ranges.	- Catalysis for hydrogen evolution reaction (HER) [74]. - Electrocatalytic applications [69].	- Exceptional catalytic efficiency due to the synergistic effects of three metals. - High durability and thermal stability. - Extended lifetime in harsh chemical environments.

**Table 6:** Comparison of optical properties and applications of different gold nanostructures.

Property	Gold Nanospheres	Gold Nanorods	Gold Nanoshells
**SPR**	Visible region (∼520–550 nm)	UV–Vis region: 500–550 nm Tunable (visible to NIR, ∼600–1200 nm)	Visible to NIR (600–1200 nm)
**Shape**	Isotropic (sphere)	Anisotropic (rod-like)	Hollow
**Applications**	- General PTT [75]. - Drug delivery, biosensing [76].	- Biosensing, Drug and Gene Delivery - Two-Photon Luminescence - Photoacoustic Imaging [77].	- Cancer photothermal therapy [78]. - Drug delivery [79].
**Advantage**	Simplicity and stability	- Tunable optical properties - Two resonance peaks	- Tunable LSPR: based on core size and shell thickness. - Enhanced photothermal effect. - Wide optical range

Royston et al. reported the use of silica-coated TMV templates for the controlled deposition of Ag, Au, Pd, and Pt nanoparticles, enabling stable and uniform nanostructure formation [55]. Next study highlighted the effectiveness of the genetically engineered Tobacco mosaic virus (TMV1Cys) as a robust template for formation of Au-Pd alloy nanoparticles synthesized using biomineralization methods by Lim et al. (2010) [51]. This method enabled the synthesis of metal nanoparticles under mild conditions without the need for high temperatures or extreme chemical processes. Another rod-shaped virus tomato mosaic virus (ToMV) where Shah et al. presented an energy-efficient method for creating nanohybrid using ToMV particles as a template. They fabricated alloys of gold (Au), platinum (Pt), and palladium (Pd) in several combinations within the ToMV's 4 nm central channel. Precise synthesis of both three-metal (AuPtPd) and two-metal (AuPt, AuPd, and PtPd) alloyed nanowires was achieved using a straightforward wet-chemical reduction process at room temperature [34]. This method proved to be highly effective in controlling the alloy composition, producing nanowires with precisely tunable properties that can be tailored for specific applications in catalysis and sensing.

**Fig. 8:**
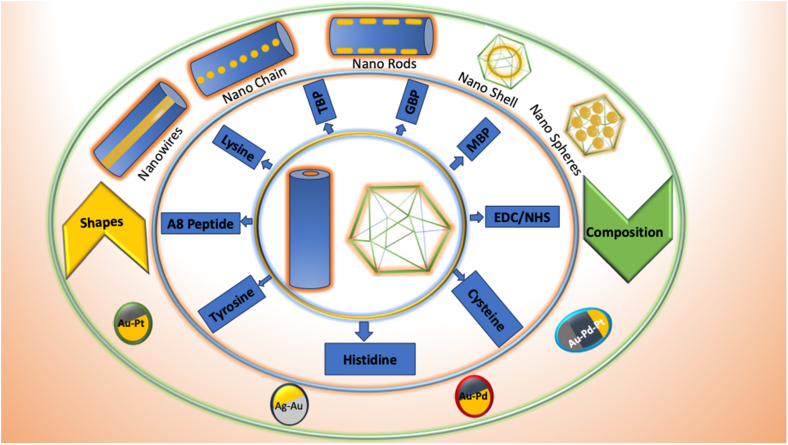
Illustrating various genetic mutations and chemical reaction to graft nanoparticle to the virus capsid, highlighting diverse shapes and compositions of nanoparticles attached to the viral structure.

Depending upon the method of synthesis, the shape of nanoparticles grafted can also be determined. Biomineralization has been employed to graft nanospheres and nanowires within the tubular cavities and sometimes to coat the entire virus capsid uniformly with metal [34,36]. So far direct grafting has offered versatility in the shapes of nanoparticles, enabling the stabilization of various forms, such as gold nanospheres [25], gold nanorods [80] and gold nanoshells [44] (Fig. 8).

Yonezawa et al. utilized thiocholine bromide-functionalized (TCB) gold nanoparticles, with TMV serving as the template for nanostructure preparation. TCB is a small cationic thiol compound that facilitates its dense packing on the negatively charged TMV surface. This electrostatic attraction enables for the spontaneous adsorption and self-fusion of nanoparticles on TMV, thus forming semi-continuous, rod-like structures at room temperature [81]. This self-assembly approach laid the groundwork for later studies exploring more precise control over nanoparticle growth within viral templates. Taking into account the strong affinity of sulfhydryl groups to gold, site-specific binding of gold species within the nanotubes of TMV was conducted by Zhou et al. using a TMV-T103C variant. This method enabled precise biomineralization of AuNPs within the TMV channels, avoiding the unspecific growth of AuNPs on the TMV exterior. This innovative approach allowed for the first controlled synthesis of AuNPs, gold nanoclusters (AuNCs), and gold nanowires inside TMV nanotubes, facilitated by the targeted mutation of threonine 103 to cysteine and a carefully optimized chemical growth process [26]. This work achieved a more controlled and selective nanoparticle growth, representing a significant advancement in nanobiomaterial synthesis within viral nanostructures*.* Durán-Meza et al. reported the formation of VLPs by self-assembling the CP of CCMV around AuNPs, including spheres, rods, and shells. All gold structures were negatively charged to bind with the CP's positive N-terminus, leading to VLP formation. The use of wild-type CCMV CPs to encapsulate both spherical and rod-shaped gold nanoparticles without ligands was uniquely demonstrated in this study, highlighting a significant advancement in nanotechnology and the versatile application of CCMV CPs in creating complex nanostructures [44].

## 6 Applications of nanohybrids-AuNPs grafted on plant viruses

The integration of gold nanoparticles (AuNPs) with plant virus scaffolds offers functional advantages that go beyond what free AuNPs can achieve alone. While AuNPs possess unique plasmonic, catalytic, and photothermal properties, their performance is often limited by issues such as poor colloidal stability, uncontrolled aggregation, and low reproducibility in surface arrangements. These limitations are overcome by Virus–AuNP hybrids. Precise control over nanoparticle spacing, density, and orientation is provided by the rigid and symmetrical architecture of virus capsids. Phenomena such as plasmon coupling, thermal conversion, and catalytic activity are critically influenced by these factors. As a result, these hybrids show superior performance in applications such as SERS, photothermal therapy, and catalysis, with improved sensitivity, stability, and reusability.

### 6.1 Bio-medical and sensing applications

**Fig. 9:**
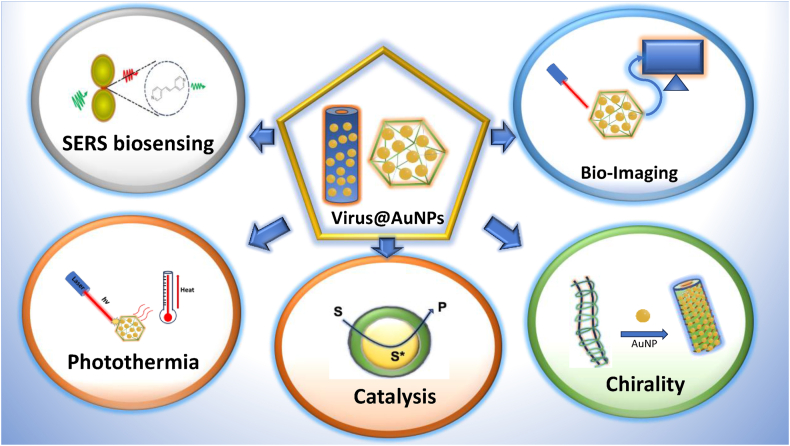
Applications of plant virus-gold nanoparticle nanohybrids across various fields. (For interpretation of the references to color in this figure legend, the reader is referred to the Web version of this article.)

As previously discussed, the shape of nanoparticles plays a critical role in determining their applications by influencing the position of plasmon bands and the stability of the nanoparticles with the virus. Below are recent examples highlighting their diverse applications (Fig. 9). Gold nanospheres, for instance, have been utilized for in solution-based biosensing [25] due to their dense assembly on the virus, which creates plasmonic hotspots in the visible region and exhibits additional plasmonic absorbance in the NIR region, making them useful for photothermal applications [30]. The efficiency of photothermal conversion is increased because the substrate facilitates the distribution and coupling of plasmonic modes more effectively than free, dispersed nanoparticles. Gold nanorods, on the other hand, have been employed in chiral sensing within the NIR spectrum [80].

Surface-enhanced Raman scattering (SERS) biosensing has significantly evolved over the years, leveraging virus-templated nanostructures to enhance detection sensitivity (Table 7). Indeed virus scaffolds enable precise spatial control over the placement and density of AuNPs, promoting the formation of plasmonic "hot spots", regions of enhanced electromagnetic field intensity. This organized architecture leads to significantly higher and more reproducible enhancement factors compared to randomly aggregated free AuNPs. In a 2016 study, AuNPs were organized in a 3D architecture by a VLP, enabling up to a tenfold enhancement in Raman signal due to efficient plasmonic hotspots [38]. Later, in 2019, AuNPs of varying sizes (5–20 nm) were grafted onto TYMV by researchers, achieving an analytical enhancement factor (AEF) up to five times greater than that of AuNPs alone [39]. By 2024, advancements using a cysteine-mutant TMV-T158C or TMV-C scaffold further improved SERS performance, demonstrating even higher enhancement factors up to 23 times for TMV-C@AuNP compared to free AuNPs, highlighting precise nanoparticle assembly and superior biosensing capability in solution [25]. These developments reflect a clear trend of virus-templated, stable, and ultrasensitive SERS biosensing platforms.

**Table 7:** Analytical enhancement factors of virus-templated nanoparticles for BPE as molecular probes.

Virus template	NP size (nm)	AEF
Cowpea Mosaic Virus [38]	24–30	∼10-fold (qualitative)
TYMV [39]	20	1 × 10^4^
TMV-C [25]	10	2.0 × 10^5^
TMV-C [25]	16	2.8 × 10^5^

The non-toxic nature of plant viruses, combined with the unique properties of gold nanoparticles, make the nanohybrids promising tools for biomedical applications. In this study, a method for detecting tumor cells using a mix of gold, TMV, and folic acid was introduced by Qu et al., exploiting the peroxidase-like activity for colorimetric detection. Au@TMV nanowires, synthesized with TMV as a template, measure 4 nm in diameter and 200–300 nm in length. Moreover, its significant aspect ratio contributes to SPR effects, enhancing thermal effects for potential applications in cancer detection and thermotherapy research [82].

Building on his previous study [37], the same principles were applied by Aljabali et al. in 2019 to use these gold-coated virus particles as computed tomography (CT) imaging contrast agents**.** The functionalized Au-CPMV particles exhibited enhanced imaging properties, demonstrating their promising applications in medical diagnostics and imaging technologies. Both studies highlight the versatility of PAH-modified CPMV as a scaffold for developing functionalized nanomaterials with diverse biomedical application potential [36]. Metavarayuth et al. demonstrated that nanotopographical cues from structurally ordered plant virus proteins, particularly using hybrid viral gold nanorods, to investigate the impact of highly ordered viral coat proteins on the osteogenic differentiation of bone marrow-derived mesenchymal stem cells (BMSCs). This effect is primarily attributed to the physical structure of the virus nanoparticles, rather than their chemical properties [83].

### 6.2 Catalysis and nanodevices

Due to the robust nature of the viruses and their nanohybrids significantly broadens their range of applications like catalysis and nanodevices etc. The viral capsid provides a confined, chemically tunable microenvironment that enhances catalytic selectivity and stability. The work by Liu et al. on AuNPs encapsulated in CCMV cages has shown significant potential as stable and recyclable heterogeneous catalysts for the reduction of nitroarenes, in contrast to bare AuNPs, which are more susceptible to aggregation or deactivation. These hybrid nanoreactors improve the stability of AuNPs and offer controlled reactivity and substrate selectivity due to the virus cages charged and size-specific properties. The reaction proceeds via a sequence from nitro group to hydroxylamine and then to amine, with the reduction rates influenced by both steric and electrostatic interactions within the protein cage. This innovative approach not only enhances the catalyst's lifetime but also allows for the reuse of the nanoparticles, providing a promising pathway for efficient and sustainable chemical processing [84]. In the study, Muller et al. focused on constructing a 3D nanostructure using Tomato Bushy Stunt Virus (TBSV) nanoparticles and nano-gold attachment. A layered virus structure is developed by taking advantage of the specific interaction between Ni-NTA conjugated with nanogold and histidine-tagged TBSV. By using this procedure, it has been possible to assembly of TBSV into bilayers, demonstrating the potential for creating intricate three-dimensional nanodevices based on viral nanoparticles [48]. Royston et al. using modified TMV templates engineered to express cysteine residues, researchers successfully assembled high-area nickel and cobalt surfaces on gold-patterned substrates. This assembly resulted in over a tenfold increase in surface area and twofold increase in electrode capacity in nickel-zinc battery systems. Electroless deposition enabled the coating of metals uniformly, thus improving durability and functionality of the electrodes. This method demonstrates significant potential for creating oriented, high-surface-area materials and advancing the integration of biologically derived components into functional nanodevices [85].

## 7 Conclusion and Perspective

In the past two decades, viral capsids have become crucial tools in nanotechnology due to their adaptability, biocompatibility, and self-assembling properties. Such stable, biocompatible, and chemically versatile capsids have been demonstrated to utilize self-assembly to encapsulate various drug cargos, embed photoactive compounds, and construct precise 3D nanoparticle frameworks that enable a variety of applications. Key uses include: (1) Biosensors with high sensitivity (2) Bioimaging with precise targeting (3) Catalysis (4) Energy harvesting (5) Plasmonic nanoparticles with tailored sizes and shapes. Leveraging the diverse morphologies of viruses and the various shapes of AuNPs has broadened the scope of potential applications.

**Fig. 10:**
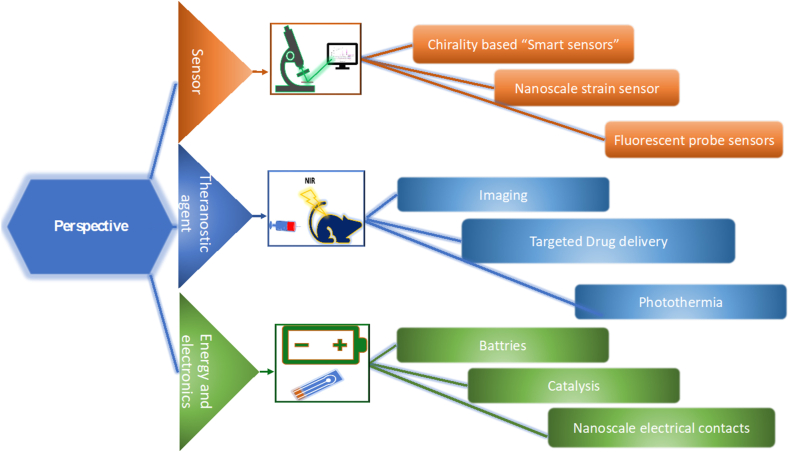
The future application of the Nanohybrids based on plant Viruses and Gold. (For interpretation of the references to color in this figure legend, the reader is referred to the Web version of this article.)

Recent advances in synthetic biology and materials science are improving viral biotemplate functionality, making them more versatile for future applications (Fig. 10). Research has shown potential in areas like nanoscale strain sensors, when deformed, the optical properties of the nanoparticles change, making them suitable for detecting mechanical deformations in soft matter [86]. The development of multifunctional materials is promising for applications that require simultaneous detection and treatment applications, particularly in cancer therapy and drug delivery. For example, optical antennas can target, image, and transport drugs directly within cells, offering an innovative approach to cellular imaging and therapeutic delivery [87], Liu et al. describes the use of CCMV as a template for creating hybrid gold nanoparticles with controlled silica deposition so that they exhibit improved stability and catalytic activity hence showing their potential in application such as catalysis, drug delivery, and bio-imaging [88]. With studies demonstrating how viral capsids like TMV can be used for unique applications, such as creating chiral metamaterials for sensing applications [89]. Furthermore, targeted growth of Au nanoparticles on the viral ends, such as nanodumbell Au-TMV-Au presents opportunities for future applications, like creating nanoscale electrical contacts [90]. There is a growing interest in grafting other nano-objects, such as carbon dots or quantum dots [31] as well which could expand the area of Fluorescence Imaging Probes and sensing realted applications. Innovations can also include the simultaneous grafting of different nanoparticles for multiple functionalities, like metal-enhanced fluorescence and SERS.

The huge, yet not fully realized potential in the study of plant viruses, particularly due to their structural diversity, which holds promise for innovative applications. The detailed structural properties and composition of plant virus capsids, along with their genetic material, are still not fully explored. Their unique morphology and structural flexibility e.g PVX virus could pave the way for designing novel nanohybrids. Use of different kinds of metal NP grafting onto these capsids could further expand applications in biomedical imaging, treatment, and emerging fields like magnetic and photothermal hyperthermia. Icosahedral viruses, such as CCMV, form biohybrid assemblies that act as nanocontainers with promising applications across biomedicine. Capsid stability can be tailored through surface chemistry for targeted drug delivery, making them powerful tools for precision medicine. As technological advances continue, the economic and industrial aspects of virus-based materials are increasingly relevant, driving the development of cost-effective production methods. Diverse shapes, nanoparticle compositions, and stability of nanohybrids are crucial to unlocking their full potential. Computational modeling in a close interplay with highly advanced characterization techniques will enable further refinement in design and functionality of nanohybrids, which would enable their continuous improvement across various applications.

However, realizing these promising applications will require addressing a range of practical and translational challenges that currently limit their widespread adoption. Challenges such as costly and resource-intensive large-scale production, batch variability, and immunogenicity remain. Overcoming these barriers will require scalable recombinant production systems, improved purification protocols, and surface modifications to enhance biocompatibility while minimizing immune recognition. Addressing regulatory hurdles, safety concerns, and standardization issues will be equally critical to enable the successful translation of these nanomaterials into clinical theranostic and drug delivery platforms.

## CRediT authorship contribution statement

**Haziq Naseer Khan:** Writing – review & editing, Writing – original draft, Conceptualization. **Nguyêt-Thanh Ha-Duong:** Writing – review & editing, Funding acquisition, Conceptualization.

## Declaration of generative AI and AI-assisted technologies in the writing process

During the preparation of this work the authors used ChatGPT in order to generate the graphical abstract. After using this service, the authors edited the content as needed and take full responsibility for the final graphical abstract.

## Declaration of Competing interest

The authors declare no conflicts of interest.

## Data Availability

Data will be made available on request.
